# Photodynamic Treatment Induces an Apoptotic Pathway Involving Calcium, Nitric Oxide, p53, p21-Activated Kinase 2, and c-Jun *N*-Terminal Kinase and Inactivates Survival Signal in Human Umbilical Vein Endothelial Cells

**DOI:** 10.3390/ijms12021041

**Published:** 2011-02-07

**Authors:** Wen-Hsiung Chan

**Affiliations:** Department of Bioscience Technology and Center for Nanotechnology, Chung Yuan Christian University, Chung Li 32023, Taiwan; E-Mail: whchan@cycu.edu.tw; Tel.: +886-3-2653515; Fax: +886-3-2653599

**Keywords:** PDT, apoptosis, calcium, NO

## Abstract

Photodynamic treatment (PDT) elicits a diverse range of cellular responses, including apoptosis. Previously, we showed that PDT stimulates caspase-3 activity, and subsequent cleavage and activation of p21-activated kinase 2 (PAK2) in human epidermal carcinoma A431 cells. In the current study, pretreatment with nitric oxide (NO) scavengers inhibited PDT-induced mitochondrial membrane potential (MMP) changes, activation of caspase-9, caspase-3, p21-activated protein kinase 2 (PAK2) and c-Jun *N*-terminal kinase (JNK), and gene expression of p53 and p21 involved in apoptotic signaling. Moreover, PAK2 activity was required for PDT-induced JNK activation and apoptosis. Inhibition of p53 mRNA expression using small interfering RNA (siRNA) additionally blocked activation of PAK2 and apoptosis induced by PDT. Importantly, our data also show that PDT triggers cell death via inactivation of ERK-mediated anti-apoptotic pathway. PDT triggers cell death via inactivation of the HSP90/multi-chaperone complex and subsequent degradation of Ras, further inhibiting anti-apoptotic processes, such as the Ras→ERK signal transduction pathway. Furthermore, we did not observe two-stage JNK activation for regulation of PAK2 activity in the PDT-induced apoptotic pathway in HUVECs, which was reported earlier in A431 cells. Based on the collective results, we have proposed a model for the PDT-triggered inactivation of the survival signal and apoptotic signaling cascade with Rose Bengal (RB), which sequentially involves singlet oxygen, Ca^2+^, NO, p53, caspase-9, caspase-3, PAK2, and JNK.

## 1. Introduction

Photodynamic treatment (PDT) of cells involves the selective delivery of a photosensitive dye into target cells, followed by visible light irradiation. Interaction of the excited photosensitizer with molecular oxygen results in the formation of reactive oxygen species (ROS), such as singlet oxygen (^1^O_2_) and hydroxyl radicals, which damage cellular constituents and are possibly responsible for triggering cell destruction [1,2]. Photosensitizers include dyes, such as Rose Bengal (RB) and methylene blue (MB), drugs such as tetracyclines and chlorpromazine, and endogenous porphyrins [3,4]. Recently, PDT has been applied to treat solid malignancies, non-malignant tumors and lung cancer [5,6]. PDT induces a diverse range of cellular responses, including apoptosis [7], which plays an important role in embryogenesis and homeostasis of multicellular organisms. Impairment of apoptosis may cause several human diseases, including neurodegenerative disorders and cancer [8]. We previously showed that PDT triggers apoptosis through multiple biochemical changes, including singlet oxygen generation as well as activation of JNK, caspase-3, and PAK2 [9,10]. These effects were blocked by the antioxidants l-histidine and curcumin, suggesting that ROS play important roles in PDT-induced apoptosis [9,10].

Nitric oxide (NO) is an important second messenger involved in a variety of cellular responses and biological functions, including tumor development, metastasis and apoptosis [11–13]. NO is largely produced in mitochondria through the actions of a Ca^2+^-sensitive mitochondrial NO synthase (NOS) [14,15]. This NOS-mediated NO production controls oxygen consumption and mitochondrial membrane potential through cytochrome c oxidase; the NO molecule is subsequently reactivated with superoxide to produce peroxynitrite, which further modifies its target substrates and induces oxidative stress [16–18]. Recent studies have shown that oxidative stress and Ca^2+^ influx act as upstream regulators of mitochondrial NOS activity [19,20].

Protein phosphorylation appears to be involved in the regulation of apoptosis. Changes in protein kinase activity observed during apoptosis in a variety of cell types [21] signify a role of phosphorylation in apoptosis control. In particular, c-Jun *N*-terminal kinase (JNK) acts as a key component in regulating entry into apoptosis in several cell types [22–24]. In addition to JNK, p21-activated kinase (PAK) may be involved in cell death signaling events during apoptosis [9,25–28]. Although the direct downstream substrates of PAKs are largely unknown, earlier studies have established that these proteins act as upstream regulators of the JNK and p38 MAPK pathways [29,30]. Furthermore, we previously reported that PAK2 activation is required for photodynamic treatment (PDT)-induced apoptosis of A431 cells [9]. However, despite accumulating evidence on the importance of PAK2 in apoptotic signaling, its direct downstream substrates and precise regulatory mechanisms remain to be elucidated.

HSP90, a 90 kDa isoform of the heat shock protein (HSP) family proteins, acts in concert with other chaperones and partners to facilitate the maturation and folding of client proteins via formation of a HSP90/multi-chaperone complex [31]. Two HSP90 client proteins, Raf-1 and MAPK/ERK kinase (MEK), are components of the Ras/extracellular signal regulated kinase (ERK)-dependent pathway that is involved in both proliferation and anti-apoptosis [32]. However, there are no documented reports on the effects of PDT on ERK-mediated survival signaling in HUVECs.

Earlier studies by our group have shown that PDT activates caspase-3 and subsequent p21-activated kinase 2 (PAK2) cleavage/activation and DNA fragmentation [9,10]. In this study, we have focused on clarifying the precise regulatory mechanisms of PDT-induced apoptosis in HUVECs.

## 2. Results and Discussion

**Figure 1.**
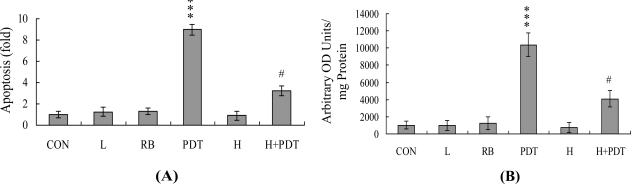
Photodynamic treatment (PDT) induces apoptosis and reactive oxygen species (ROS) generation of HUVECs. HUVECs were incubated with or without Rose Bengal (5 μM; RB) and/or l-histidine (500 μM; H) in the dark at 37 °C for 1 h. Cells were treated with visible light (L) for 30 min or left untreated, followed by incubation in the absence of light at 37 °C for a further 12 h. (**A**) Apoptosis was detected with the Cell Death Detection ELISA kit; (**B**) ROS generation was assayed using DCF-DA (20 μM) dye. Values are presented as means ± SEM of six determinations. *** *P* < 0.001 *versus* the untreated control group (CON); ^#^ *P* < 0.001 *versus* the PDT-treated group.

Singlet oxygen is an important cellular mediator for PDT-induced responses [33–35]. We have already demonstrated that singlet oxygen mediates the PDT-induced activation of caspase-3 and subsequent apoptotic biochemical changes in A431 cells [9,10]. However, the cytotoxic effects of PDT on HUVECs have not been established to date. Experiments in the current study showed that PDT induces apoptosis and ROS generation in HUVEC cells. Moreover, these biochemical events are effectively blocked upon pretreatment with l-histidine, a singlet oxygen-specific scavenger (Figure 1A, B), confirming that PDT triggers apoptosis of HUVECs via singlet oxygen generation.

**Figure 2.**
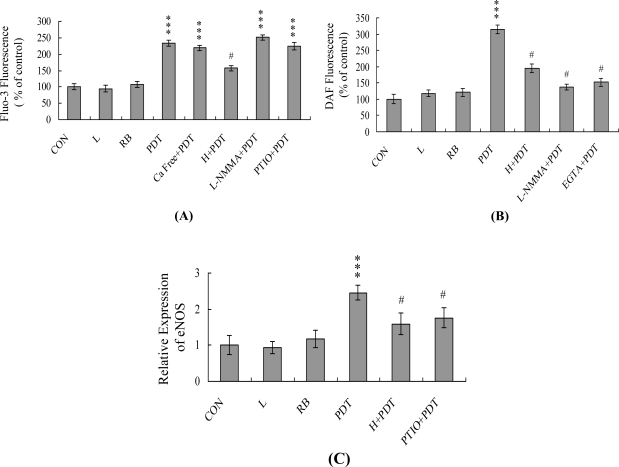
PDT triggers changes in the intracellular calcium and nitric oxide (NO) content in HUVECs. HUVECs were incubated with or without Rose Bengal (5 μM; RB), l-histidine (500 μM; H), L-NMMA (400 μM), PTIO (20 μM), and EGTA (500 μM) in the dark at 37 °C for 1 h. Cells were treated with visible light for 30 min or left untreated, and incubated in the absence of light at 37 °C for a further 12 h. (**A**) Intracellular Ca^2+^ level changes were measured via estimation of intracellular Fluo-3 fluorescence intensity; (**B**) Intracellular NO generation was measured using the DAF-2DA fluorescence dye; (**C**) The mRNA levels of endothelial cell nitric oxide synthase (eNOS) were analyzed using real-time PCR. Data are presented as percentage or fold, compared to the control group (CON). *** *P* < 0.001 *versus* the untreated control group; ^#^ *P* < 0.001 *versus* the PDT-treated group.

Changes in [Ca^2+^]i in HUVECs subjected to PDT were detected using the Fluo-3AM fluorescence dye. Cells cultured in Ca^2+^-containing medium showed a ∼2.2-fold increase in [Ca^2+^]i following PDT, whereas this increase had no effect in treated cells cultured in Ca^2+^-free medium (Figure 2A). These findings indicate that the rise in [Ca^2+^]i is primarily attributed to calcium release from intracellular stores, such as those found in the endoplasmic reticulum, mitochondria, nucleus and/or calcium-binding proteins (Figure 2A). In addition, L-NMMA, an inhibitor of NOS, and PTIO, an inhibitor of NOS and scavenger of NO, had no effect on the PDT-induced [Ca^2+^]i increase, whereas pretreatment with l-histidine led to a significant decrease in [Ca^2+^]i (Figure 2A). The results suggest that elevation of [Ca^2+^]i induced by PDT is regulated by singlet oxygen generation, but not NO. Experiments using the NO-sensitive dye, DAF-2DA, to measure intracellular NO generation during PDT-induced apoptosis revealed increased NO levels in HUVECs (Figure 2B). However, this increase was prevented by pretreatment of cells with the NOS inhibitor, L-NMMA, or the Ca^2+^ chelator, EGTA (Figure 2B). Real-time RT-PCR analyses disclosed significant upregulation of endothelial cell nitric oxide synthase (eNOS) in HUVECs exposed to PDT, which was effectively blocked upon pretreatment with l-histidine or PTIO (Figure 2C). These results suggest that intracellular Ca^2+^ levels play an important role in NOS activation and NO increase observed in HUVECs subjected to PDT.

**Figure 3.**
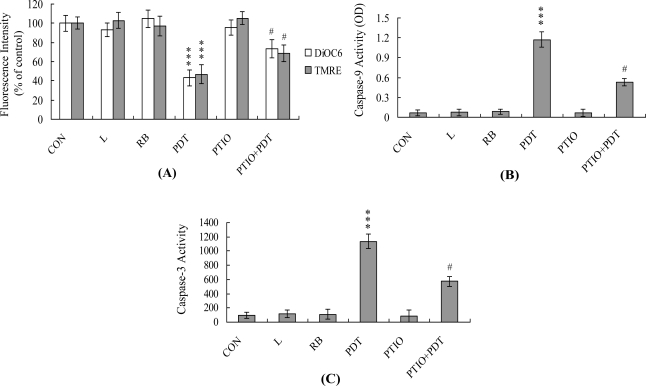
PDT induces mitochondrial membrane potential (MMP) changes and caspase activation in HUVECs. HUVECs were pre-treated with or without PTIO (20 μM) for 1 h and exposed to PDT or left untreated. (**A**) To examine MMP changes, cells were incubated with 40 nM DiOC6(3) or 1 μM TMRE at 37 °C for 1 h, and analyzed using spectrofluorometry; (**B**) Caspase-9 activity was assayed using the Colorimetric Caspase-9 Assay kit; (**C**) Caspase-3 activity was analyzed using Z-DEVD-AFC as the substrate. Values are presented as means ± SEM of eight determinations. *** *P* < 0.001 *versus* the untreated control group (CON); ^#^ *P* < 0.001 *versus* the PDT-treated group.

Mitochondrial membrane potential (MMP) changes are directly associated with apoptosis [36–38], and MMP changes observed in PDT-exposed cells [39]. Examination of the effects of PDT on MMP changes in HUVECs revealed suppression of uptake of DiOC6(3) and TMRE into mitochondria, indicative of significant MMP loss (Figure 3A). We additionally monitored activation of caspases-9 and -3, which are involved in MMP change-mediated apoptosis. Photodynamic treatment of HUVECs stimulated the activities of both caspases-9 (Figure 3B) and -3 (Figure 3C). Notably, both MMP loss and caspase activation were significantly inhibited upon incubation of cells with 20 μM PTIO prior to PDT (Figure 3A–C). Our results indicate that NO acts as an upstream regulator for loss of MMP and activation of caspases-9 and -3 during PDT-induced apoptosis of HUVECs.

**Figure 4.**
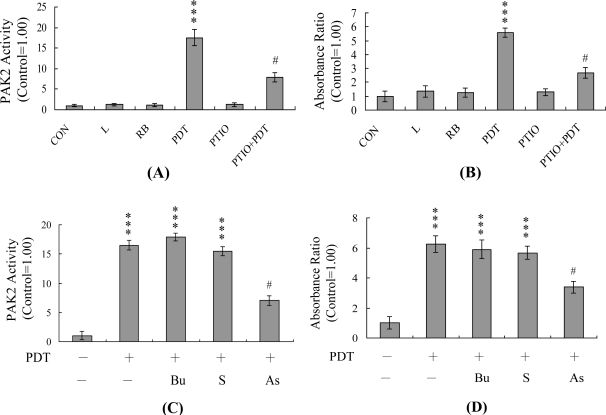

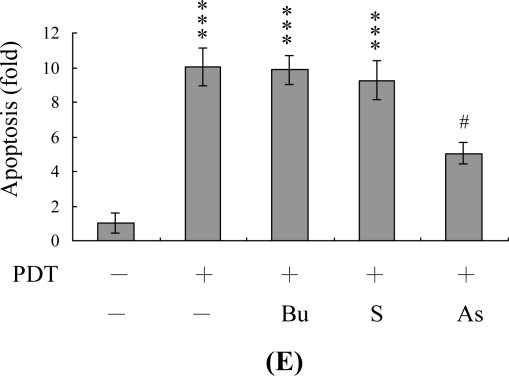
PAK2 and JNK are activated during PDT-induced apoptosis of HUVECs. HUVECs were pre-treated with or without PTIO (20 μM) for 1 h, followed by PDT. After treatment, cells were further incubated in the absence of light at 37 °C in a CO_2_ incubator for 12 h. (**A**) PAK2 was immunoprecipitated and kinase activities assayed using myelin basic protein as the substrate; (**B**) Cell extracts (60 μg) were analyzed for JNK/AP-1 activity with the ELISA kit; (**C**–**E**) Cells were transfected with buffer (Bu), sense (S) or anti-sense (AS) oligonucleotides for 72 h, and subjected to PDT. PAK2 activity (C), JNK activity (D), and cell apoptosis (E) were assessed. *** *P* < 0.001 *versus* the untreated control group (CON); ^#^ *P* < 0.001 *versus* the PDT-treated group.

In view of previous findings that p21-activated protein kinase 2 (PAK2) and JNK are activated during apoptosis and subsequently involved in apoptotic signaling events in PDT-exposed A431 [9,27,28], we investigated the involvement of PAK2 and JNK in PDT-triggered apoptosis of HUVECs. Immunoprecipitation kinase activity assays revealed PAK2 and JNK activation in PDT-exposed HUVECs, which were effectively prevented by pre-treatment with PTIO (Figure 4A, B). To further establish the relationship between PAK2 and JNK activation as well as the specific function of PAK2 in PDT-induced apoptosis, we incubated HUVECs with anti-sense or sense oligonucleotides against PAK2 for three days, subjected the cells to PDT, and analyzed PAK2 activity via immunoprecipitation. Pre-incubation of HUVECs with an anti-sense oligonucleotide against PAK2 led to a significant decrease in the PAK2 level (by ∼40%), compared to control cells exposed to PDT, whereas the sense oligonucleotide had no such effect (Figure 4C). This decreased PAK2 activation was associated with significant decreases in PDT-induced activation of JNK and apoptosis (Figure 4D, E). Thus, our findings strongly suggest that PAK2 plays an important role in PDT-induced JNK activation and apoptosis of HUVECs.

**Figure 5.**
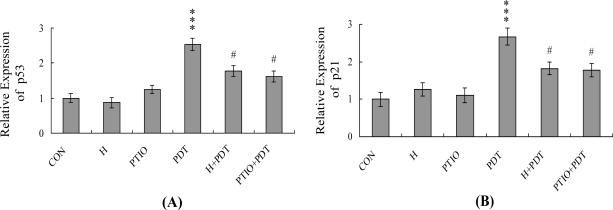
Effects of l-histidine and PTIO on the mRNA expression levels of p53 and p21. HUVECs were pre-incubated with or without l-histidine (500 μM; H) and PTIO (20 μM) for 1 h, followed by PDT. The mRNA levels of p53 (**A**) and p21 (**B**) were analyzed using real-time PCR. The given values are representative of five independent determinations. *** *P* < 0.001 *versus* the untreated control group (CON); ^#^ *P* < 0.001 *versus* the PDT-treated group.

**Figure 6.**
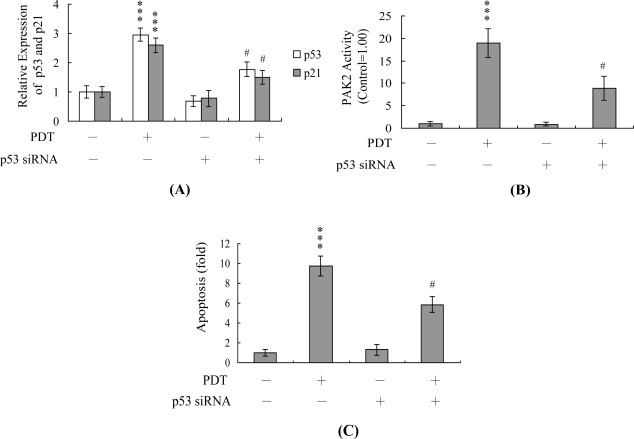
Knockdown of p53 protects HUVECs against PDT-induced apoptosis. HUVECs were transfected with siRNA targeting p53, incubated for 24 h, and subjected to PDT. (**A**) The p53 and p21 mRNA levels were analyzed using real-time PCR; (**B**) PAK2 activities were assayed using myelin basic protein as the substrate; (**C**) Cell apoptosis was measured as described in Figure 1. *** *P* < 0.001 *versus* the untreated control group; ^#^ *P* < 0.001 *versus* the PDT-treated group.

Real-time RT-PCR analyses disclosed significant upregulation of p53 and p21 mRNA levels in HUVECs subjected to PDT. Moreover, these changes were blocked upon pretreatment with l-histidine or PTIO (Figure 5A, B). To further ascertain the roles of p53 and p21 in PDT-induced apoptosis, we used targeted siRNAs to suppress p53 expression in HUVECs, followed by photodynamic treatment of cells and testing for viability. The p53 siRNA-mediated knockdown led to a significant decrease in p53 and p21 mRNA expression in PDT-exposed HUVECs (Figure 6A). These decreases in p53 and p21 mRNA were associated with a significant reduction in PDT-induced activation of PAK2 (Figure 6B) and apoptosis (Figure 6C). Based on these findings, we suggest that PDT upregulates p53 and p21 expression levels in HUVECs, which contribute to subsequent apoptosis of treated cells.

**Figure 7.**
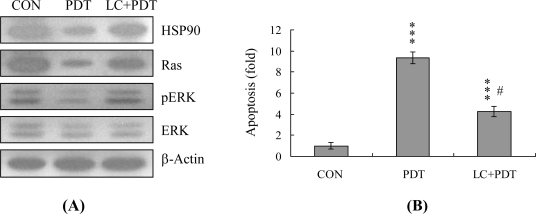
Effect of PDT on HSP90 and components of survival signaling. HUVECs were incubated with or without lactacystin (LC; 10 μM) for 1 h, and subjected to PDT. (**A**) The protein levels of HSP 90, Ras and ERK-1/2, and phosphorylation of ERK-1/2 were evaluated. β-actin was used as the loading control; (**B**) Cell apoptosis was detected with the TUNEL assay. Data are representative of five independent experiments. *** *P* < 0.001 *versus* untreated control group (CON); ^#^ *P* < 0.001 *versus* PDT-treated group.

A recent study by our group further showed that additional regulatory mechanisms are involved in PDT-induced apoptosis in HUVECs. Caraglia *et al.* reported that survival signaling processes protect against apoptosis induced by specific stimuli [40,41]. Furthermore, some apoptotic stimuli inhibit the Ras→ERK survival signal pathway by suppressing the protein levels of various survival components [41–43]. Experiments from the current study disclosed that PDT triggers a decrease in the HSP90 and Ras protein levels and inhibits ERK activity (Figure 7A). Inhibition of protein expression and activities of survival signal components by PDT was effectively blocked upon pretreatment with lactacystin, a specific proteasome inhibitor (Figure 7A). Moreover, lactacystin prevented PDT-induced apoptosis in correlation with blockage of the decrease in survival signal components (Figure 7A, B). Our data suggest that Ras proteins are degraded via proteasome-dependent pathways in PDT-treated HUVECs, subsequently leading to reduction of ERK activity for apoptosis.

In recent years, PDT has emerged as a promising therapeutic protocol for treatment of malignant and non-malignant diseases [5]. Previous studies by our group have shown that PDT elicits singlet oxygen formation in A431 cells, and singlet oxygen scavengers prevent PDT-induced caspase-3 cleavage/activation and subsequent PAK2 activation [9,10]. In addition, PDT has been shown to induce cancer cell apoptosis via the Ca^2+^ and NO-mediated signal pathway [14]. In the present study, we have further clarified the regulatory roles of singlet oxygen, NO, calcium, p53, PAK2 and JNK in PDT-induced apoptosis of HUVECs.

Intracellular calcium levels play an important role in regulating cell death [14,44,45]. In the present study, we examined whether PDT induces apoptosis of HUVECs through intracellular calcium increase. We observed increases in [Ca^2+^]i following PDT, which were largely attributed to influx of Ca^2+^ from intracellular Ca^2+^ storage organelles (Figure 2). This effect was significantly blocked by l-histidine (Figure 2), indicating that PDT-induced singlet oxygen generation is responsible for the elevation of intracellular calcium concentrations in PDT-treated HUVECs.

NO is an endogenous product generated from the catalysis of NADPH, O_2_ and l-arginine by nitric oxide synthase. A recent study demonstrated that NO is involved in apoptosis triggered by several different types of stimuli [14,15]. The regulatory actions of NO on the mitochondrial apoptotic signaling pathways are well documented. Decreased ratios of Bcl-2/Bax and inhibition of electron transport are among the regulatory mechanisms of NO-mediated apoptosis [46]. Decreased Bcl-2/Bax ratio and impairment of mitochondrial electron transport may trigger loss of MMP and release of cytochrome c, which are involved in the control of apoptosis. In addition, tamoxifen treatment increases intramitochondrial Ca^2+^ concentrations, leading to stimulation of mitochondrial NO synthase activity and NO production in rat livers and human breast cancer MCF-7 cells [15]. Here, we observed NO generation following PDT of HUVECs, with ∼3.2-fold higher intracellular NO levels in treated cells *versus* untreated controls (Figure 2B). Pretreatment with EGTA significantly prevented this increase in intracellular NO (Figure 2B), implying that NO production in PDT-treated HUVECs is dependent on the intracellular calcium concentration. Furthermore, consistent with the finding that NOS is the main source of NO during stimulus-triggered apoptosis [14,15], we observed ∼2.5-fold higher eNOS mRNA in PDT-treated HUVECs *versus* untreated controls (Figure 2C). The regulatory role of NO in apoptosis is complex, and NO-mediated apoptotic effects are modulated via distinct mechanisms in different cell types [13,47]. NOS substrates and NO donors inhibit photodynamic treatment-induced apoptosis in CCRF-CEM cells [48]. In the current study, PTIO treatment attenuated MMP loss and decreased caspase activation (Figure 3A–C), suggesting that NO generation is an important mediator of apoptosis in PDT-exposed HUVECs.

Recently, our laboratory and other researchers showed that PAK2 is a target substrate for caspases activated by various apoptotic stimuli, including anti-Fas antibodies, tumor necrosis factor-α, ceramide and environmental stress factors, such as UV irradiation, heat shock and hyperosmotic shock [26–28,49]. Activation of PAK2 appears to be a critical step for stimulation of JNK activity during PDT-induced apoptosis [9,10]. However, the functional role of caspase-generated activation of PAK2 remains obscure at present. In previous experiments, apoptosis was delayed upon transfection of dominant-negative PAK2 (full-length or *N*-terminally truncated) into CHO cells stably expressing a CD4-Fas chimera [26]. Similarly, microinjection of active PAK into early frog embryos caused cleavage arrest [50,51]. Recent studies further demonstrated that an anti-activated PAK2 polyclonal antibody recognizes several phosphoproteins in mitotic HeLa and A431 cells, including lamins A and C [52]. PAKs are significantly involved in cell cycle control. Our group recently showed an association of decreased PAK2 protein expression and activation with significant inhibition of methylglyoxal-induced apoptosis in human osteoblasts [53], strongly suggesting that PAK2 plays an important role in apoptosis triggered by methylglyoxal. Data from the current study demonstrate that PAK2 is an important upstream regulator of JNK activation in PDT-directed apoptosis of HUVECs (Figure 4). JNK plays critical roles in apoptosis [23,24]. Based on the results, we propose that PDT triggers singlet oxygen generation and caspase-3 activation, in turn, inducing PAK2 and sequential activation of JNK, and ultimately, apoptosis.

NO-mediated apoptotic processes are associated with p53 gene activation, which is essential for regulation of the cell cycle and/or apoptotic signaling occurring through p21^Waf1/Cip1^ or Bax [54,55]. In our current study, p53 and p21 mRNA levels were upregulated upon treatment with PDT, and these increases were blocked upon pretreatment with PTIO (Figure 5A,B). Furthermore, siRNA-mediated knockdown of p53 mRNA expression prevented the PDT-induced increase in p21 mRNA and PAK2 activation, and led to a decrease in subsequent apoptosis (Figure 6A–C). These results indicate that genes encoding p53 and p21 are activated during PDT-induced apoptosis of HUVECs.

Heat shock proteins (HSP) protect proteins against proteasome- and ubiquitin-dependent degradation [56,57]. HSP90, the most abundant molecular chaperone protein in the intracellular system, is involved in maintaining the correct conformations of intracellular proteins and kinases, such as Raf-1 [58,59], which regulates cell proliferation and survival. Data from the present study showed an association between PDT-induced apoptosis and reduced expression of survival components, such as Ras. Inactivation of ERK-1 and ERK-2 and pretreatment with lactacystin, a proteasome inhibitor, prevented the PDT-mediated decrease in protein expression or activity (Figure 7A). Moreover, PDT suppressed HSP90 expression, thus promoting the degradation of client proteins (Figure 7A). Accordingly, we hypothesize that the PDT-induced reduction of HSP90 stimulates Ras targeting for degradation, leading to its downregulation and changes in the related signal pathways.

Importantly, previous studies have shown that PDT triggers cell apoptosis through the Ca^2+^ and NO-mediated signal pathway. Moreover, our group has demonstrated that singlet oxygen-mediated JNK activation is required for caspase and PAK2 activities that further elicit second-stage activation of JNK to trigger PDT-induced apoptosis [9,14]. The present report reveals for the first time that PDT triggers apoptosis through Ca^2+^ influx and NO production to affect P53 protein expression, and causes sequent apoptotic biochemical changes, including loss of MMP as well as activation of caspases, PAK2 and JNK in HUVEC cells. Furthermore, we did not observe two-stage JNK activation for regulation of PAK2 activity in the PDT-induced apoptotic pathway in HUVECs, which was reported earlier in A431 cells [9]. The results clearly indicate that PDT induces apoptosis through different mechanisms, depending on cell type.

## 3. Experimental Section

### 3.1. Chemicals

3-(4,5-dimethylthiazol-2-yl)-2,5-diphenyltetrazolium bromide (MTT), 2-phenyl-4,4,5,5-tetramethylimidazoline-1-oxyl-3-oxide (PTIO), l-histidine, 2′,7′- dichlorofluorescein diacetate (DCF-DA) and goat anti-rabbit immunoglobulin G (IgG) antibodies conjugated with alkaline phosphatase were purchased from Sigma (St. Louis, MO, U.S.). Anti-p53, anti-p21, anti-JNK1 (C17), anti-p-JNK (G-7) and anti-β-actin antibodies were from Santa Cruz Biotechnology (Santa Cruz, CA, U.S.). Z-DEVD-AFC was obtained from Calbiochem (La Jolla, CA, U.S.). The CDP-Star^TM^ chemiluminescent substrate for alkaline phosphatase was acquired from Boehringer Mannheim (Mannheim, Germany).

### 3.2. Cell Culture and PDT

Human umbilical vein endothelial cell (HUVEC) strain (ECV-304) was obtained from the ATCC, and the cells were cultured at 37 °C in a CO_2_ incubator in M_199_ medium containing 10% fetal calf serum, with medium changes every 24 h. For PDT, cells were incubated in medium containing 5 μM Rose Bengal (RB) in the dark for 30 min at 37 °C, followed by irradiation with a commercially available 120 W lamp from a fixed distance of 30 cm for 30 min. Cells were incubated in the absence of light at 37 °C in a CO_2_ incubator for another 12 h. The spectral output of the light source and fluence rate at the surface of cultures in the visible light region were as reported previously by our group [9]. PDT treatment conditions were similar for all the experiments performed in this study. During all treatment periods, cells were incubated with medium containing 5 μM RB. Cells were then washed twice with ice-cold PBS and lysed on ice for 10 min in 400 μL lysis buffer (20 mM Tris-HCl at pH 7.4, 1 mM EDTA, 1 mM EGTA, 1% Triton X-100, 1 mM benzamidine, 1 mM phenylmethylsulfonyl fluoride, 50 mM NaF, 20 μM sodium pyrophosphate and 1 mM sodium orthovanadate). Cell lysates were sonicated on ice for 3 × 10 s, then centrifuged at 15,000 × g for 20 min at 4 °C, and the supernatants used as cell extracts.

### 3.3. Apoptosis Assay

Oligonucleosomal DNA fragmentation in apoptotic cells was measured using the Cell Death Detection ELISA^plus^ kit according to the manufacturer’s protocol (Roche Molecular Biochemicals). Cells (1 × 10^5^) were treated with or without PDT for 30 min at 37 °C. Spectrophotometric data were obtained using an ELISA reader at a wavelength of 405 nm.

### 3.4. ROS Assay

ROS were measured in arbitrary units using 2′,7′-dichlorofluorescein diacetate (DCF-DA) dye. Cells (1.0 × 10^6^) were incubated in 50 μL PBS containing 20 μM DCF-DA for 1 h at 37 °C, and subsequently exposed to PDT. Cells were further incubated in the absence of light at 37 °C in a CO_2_ incubator for 12 h, and the relative ROS units determined using a fluorescence ELISA reader (excitation 485 nm, emission 530 nm). An aliquot of the cell suspension was lysed, the protein concentration was determined, and the results were expressed as arbitrary absorbance units/mg protein.

### 3.5. Detection of Intracellular Calcium Concentration ([Ca^2+^]i)

The [Ca^2+^]i was detected with Fluo-3 AM fluorescence dye, using a modification of the previously reported method [14,60]. Briefly, HUVECs were PDT treated, harvested and washed, and then loaded with 6 μM Fluo-3 AM in standard medium (140 mM NaCl, 5 mM KCl, 1 mM MgCl_2_, 5.6 mM glucose, 1.5 mM CaCl_2_, and 20 mM Hepes, pH of 7.4). After 30 min, the cells were washed 3 times with PBS and then resuspended in standard medium or Ca^2+^-free standard medium. The fluorescence intensity of Fluo-3 was determined using a fluorescence spectrophotometer (Hitachi, F-2000; excitation at 490 nm, emission at 526 nm).

### 3.6. Detection of Intracellular NO Content

The DAF-2DA fluorescence dye was used to detect intracellular NO, according to a modification of the previously reported method [14,61]. Briefly, treated or control cells were collected and washed, and then incubated with 3 μM DAF-2DA. After 60 min, the cells were washed 3 times with PBS and the fluorescence intensity was measured by a fluorescence spectrophotometer (Hitachi, F-2000; excitation at 485 nm, emission at 515 nm).

### 3.7. Detection of Mitochondrial Membrane Potential (MMP)

HUVECs were plated and grown on 96-well plates for 24 h, and then treated by PDT. The cells were then separately exposed to the fluorescent dyes, DiOC6(3) (40 nM/well) or TMRE (1 μM/well), for 15 min, and fluorescence was measured with a plate spectrofluorometer (excitation: 485 nm (DiOC6(3)), 535 nm (TMRE); Emission: 535 nm (DiOC6(3)), 590 nm (TMRE)).

### 3.8. Caspase Activity Assays

Caspase-9 activity was assayed using the Colorimetric Caspase-9 Assay Kit (Calbiochem, CA, U.S.). Caspase-3 activity was measured using the Z-DEVD-AFC fluorogenic substrate, as previously described [62,63].

### 3.9. Immunoprecipitation and PAK2 Activity Assay

The PAK2 antibody was prepared in our laboratory as described previously [64]. In brief, the anti-PAK2 (C15) antibody was produced in rabbits using the antigen peptide, TPLIMAAKEAMKSNR, which corresponds to *C*-terminal residues 510–524 of the human and rabbit PAK2 sequences [65,66]. A cysteine residue was added to the N-terminus to facilitate coupling of the peptide to keyhole limpet hemocyanin, as previously described [67]. Glutaraldehyde was used as the cross-linker. The anti-peptide antibody was produced and affinity purified as previously described [64]. Before immunoprecipitation, cell extracts were diluted to equal protein concentrations (1.0 mg/mL) with cell lysis solution. For immunoprecipitation of the *C*-terminal catalytic fragment of PAK2, 0.5 mL of cell extract was incubated with 10 μL of anti-PAK2 (C15) antibody (200 μg/mL) at 4 °C for 1.5 h, and then further incubated with 40 μL of Protein A-Sepharose CL-4B (30%, v/v) for 1.5 h with shaking. The immunoprecipitates were collected by centrifugation, washed three times with 1 mL of Solution A (20 mM Tris/HCl, pH 7.0, and 0.5 mM DTT) containing 0.5 M NaCl, and resuspended in 40 μL of Solution A. For measurement of PAK2 activity, the immunoprecipitates were incubated in a 50 μL mixture containing 20 mM Tris/HCl, pH 7.0, 0.5 mM DTT, 0.2 mM [γ-p^32^]ATP, 20 mM MgCl_2_ and 0.1 mg/mL myelin basic protein (MBP) at room temperature for 10 min with shaking. For determination of ^32^P incorporation into the MBP protein, 20 μL of each reaction mixture was spotted onto Whatman P81 paper (1 × 2 cm), which was then washed with 75 mM phosphoric acid and processing as previously described [68].

### 3.10. JNK Assays

JNK activity, as assayed by the presence of phosphorylated c-Jun protein, was analyzed with the AP-1/c-Jun ELISA kit, according to the manufacturer’s protocol (Active Motif, Carlsbad, CA, U.S.). Briefly, AP-1 heterodimeric complexes were collected from cellular nuclear extracts by binding to a consensus 5′-TGA(C/G)TCA-3′ oligonucleotide coated on a 96-well plate. The phospho-c-Jun was assayed using a phospho-c-Jun primary antibody and a secondary horseradish peroxidase-conjugated antibody in a colorimetric reaction.

### 3.11. Inhibition of PAK2 by Anti-sense Oligonucleotides

PAK2 sense (5′-ATC ATG TCT GAT AAC GGA GAA) and anti-sense (5′-TTC TCC GTT ATC AGA CAT GAT) oligonucleotides, representing amino acids −1 to +7 of human PAK2, were obtained from Life Technologies (Grand Island, NY). The oligonucleotides were synthesized under phosphorothioate-modified conditions, purified by HPLC, and dissolved in 30 mM HEPES buffer, pH 7.0. For transfections, cells grown in 60 mm culture dishes were incubated at 37 °C in 1 mL of Opti-MEM I medium (modified Eagle’s minimum essential medium buffered with HEPES and sodium bicarbonate), containing lipofectAMINE4 (12 μg) and oligonucleotides (70 μM) for 72 h (all reagents from Life Technologies, Grand Island, NY, U.S.). The cells were then subjected to PDT, and the cell extracts were analyzed as described above.

### 3.12. Real-Time RT-PCR Assay

Total RNA was extracted with the TRIzol reagent (Life Technologies) and purified with an RNeasy Mini kit (Qiagen), according to the manufacturers’ protocols. Real-time PCR was carried out with an ABI 7000 Prism Sequence Detection System (Applied Biosystems). The β-actin mRNA levels were quantified as an endogenous control, and used for normalization. The primers used for PCR were as follows: eNOS, 5′-CCT TTG CTC GTG CCG TGG AC-3′ and 5′-GCC CTC GTG GAC TTG CTG CTT-3′; p53, 5′-CCC ATC CTC ACC ATC ATC AC-3′ and 5′-GTC AGT GGG GAA CAA GAA GTG-3′; p21, 5′-GCC GAA GTC AGT TCC TTG TGG A-3′ and 5′-GTG GGC GGA TTA GGG CTT-3′.

### 3.13. siRNA Knockdown

Lipofectamine was used to transfect HUVECs with 150 nM of siRNA for targeting against p53 (5′-GACUCCAGUGGUAAUCUACTT-3′; sip53), or a scrambled control duplex (5′-GCGCGCUUUGUAGGAUUCG-3′; siScr). Twenty-four hours post-transfection, fresh culture medium was added, and the cells were treated by PDT.

### 3.14. Immunoblots

Immunoblotting was performed essentially as described in a previous report by our group [69]. Briefly, proteins were resolved by SDS-PAGE, transferred to PVDF membranes, and immunoblotted with anti-HSP 90, anti-Ras, anti-phospho ERK1/2 and anti-ERK1/2 antibodies (0.25 μg/mL). Proteins of interest were detected with secondary alkaline phosphatase-conjugated goat anti-rabbit or anti-mouse IgG antibodies, and visualized using the CDP-Star^TM^ chemiluminescent substrate, according to the manufacturer’s protocol.

### 3.15. Statistics

Data were analyzed using one-way ANOVA, and differences were evaluated using a two tailed Student’s t-test and analysis of variance. *P* < 0.05 was considered significant.

## 4. Conclusions

**Figure 8.**
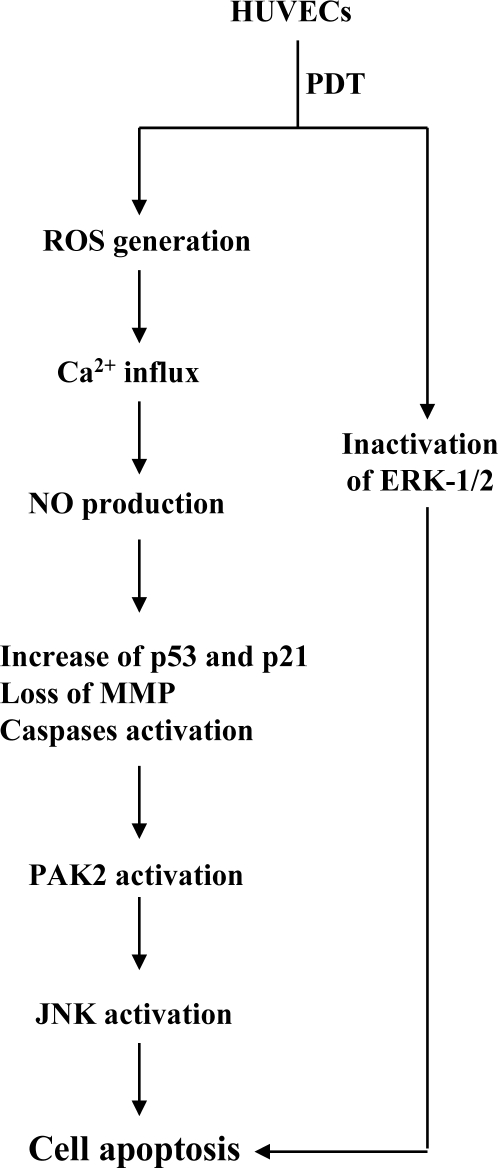
Scheme of events occurring during PDT-induced HUVEC cell death.

Based on the present findings, we have proposed a model of the PDT-induced apoptotic signaling pathways in HUVEC cells (Figure 8).
